# ^18^Fluorine-sodium fluoride positron emission tomography to evaluate arterial calcification in patients with chronic kidney disease: a pilot study

**DOI:** 10.1007/s10554-025-03553-0

**Published:** 2025-11-13

**Authors:** Aspan M. Shokrekhuda, Rylen Stratford, Wenzhu Mowrey, Na Song, Kexin Zhang, Sara Saini, Mario T. Di Dea, Gabriel Duah, Zahra Karimi, Nicholas E. S. Sibinga, Yonatan Schwartz, Jeffrey M. Levsky, Marc R. Dweck, Renee Moadel, Lionel S. Zuckier, Mark I. Travin, Wei Chen

**Affiliations:** 1 Department of Radiology, Nuclear Medicine Division, Montefiore Medical Center, Bronx, NY USA; 2 Department of Epidemiology and Population Health, Albert Einstein College of Medicine, Bronx, NY USA; 3 Department of Medicine, Nephrology Division, Albert Einstein College of Medicine, Bronx, NY USA; 4 Department of Medicine, Cardiology Division, Albert Einstein College of Medicine, Bronx, NY USA; 5 Department of Developmental and Molecular Biology, Albert Einstein College of Medicine, Bronx, NY USA; 6 Wilf Family Cardiovascular Research Institute, Albert Einstein College of Medicine, Bronx, NY USA; 7 Department of Radiology, Thoracic Radiology, Montefiore Medical Center, Bronx, NY USA; 8 BHF Centre for Cardiovascular Science, University of Edinburgh, Edinburgh, UK; 9 Department of Radiology, Nuclear Medicine Division, Columbia University Vagelos College of Physicians and Surgeons, New York, NY USA; 10 Department of Medicine, Nephrology Division, Montefiore Medical Center, NY Bronx, USA; 11 Department of Radiology, Holy Name Medical Center, NJ Teaneck, USA

**Keywords:** Arterial calcification, ^18^F-NaF PET, Chronic kidney disease, Micro-calcification, Aortic calcification

## Abstract

^18^Fluorine-Sodium Fluoride Positron Emission Tomography (^18^F-NaF PET) allows for the detection of arterial micro-calcification, but its data on people with chronic kidney disease (CKD) is limited. This pilot study aimed to determine the optimal time to perform ^18^F-NaF PET static scan and to characterize arterial macro- and micro-calcification in CKD. In 7 patients with CKD stages 3b-4 and 3 non-CKD individuals, following the injection of ^18^F-NaF, an electrocardiogram-gated chest CT, a 30-min dynamic PET, and three 10-min static PET were acquired. Radiopharmaceutical uptake was quantified for regions including the lumbar spine and 4 aortic segments. Arterial macro-calcification was quantified using Agatston scoring and micro-calcification using tissue-to-background ratio (TBR; reference: right atrium). The standardized uptake value (SUV) in lumbar spine reached a steady state ~ 60 min after injection and was comparable at each time point between CKD and non-CKD (p: 0.58–0.92). Among CKD participants, 6 had macro-calcification in coronary arteries. Abdominal aorta had the greatest macro-calcification, and ascending aorta had the lowest TBR_peak_. In the ascending aorta, macro-calcification negatively correlated with TBR_peak_ (rho = − 0.86, p = 0.01). In descending thoracic aorta, TBR_peak_ negatively correlated with serum calcium (rho = − 0.81, p = 0.03), and positively correlated with parathyroid hormone (rho = 0.93, p = 0.003) and thrombomodulin (rho = 0.79, p = 0.04). The optimal time for ^18^F-NaF PET static scan is ~ 60 min for people with and without CKD. Evaluation of macro- and micro-calcification in aorta revealed preliminary spatial patterns and their associated serum markers. Our findings help to establish technical specifications for image acquisition and analyses of ^18^F-NaF PET/CT in CKD.

**Supplementary Information:**

The online version contains supplementary material available at 10.1007/s10554-025-03553-0.

## Introduction

Arterial calcification is pathological deposition of calcium and phosphate in the form of calcium hydroxyapatite in the arterial walls. It is associated with arterial stiffness and systolic hypertension and ultimately contributes to high mortality [1, 2]. Based on the size of calcification nodules, arterial calcification can be classified into macro-calcification (i.e., calcification nodules ≥50 μm) and micro-calcification (< 50 μm). It is crucial to assess both arterial macro- and micro-calcification because they have distinct biological activities and associated cardiovascular risks [3]. Whereas macro-calcification stabilizes atherosclerotic plaques, micro-calcification represents an early and active stage of calcification and inflammation and has been associated with plaque rupture [4–8].

Computed tomography (CT) can detect arterial macro-calcification but it lacks the sensitivity to detect micro-calcification. Positron emission tomography (PET) using ^18^Fluorine-sodium fluoride (^18^F-NaF) has emerged as an innovative imaging technique to detect micro-calcification in arteries [9]. ^18^F-NaF binds to the surface of calcium hydroxyapatite such as bone by exchanging hydroxyl ions with fluoride to form fluorapatite and preferentially binds to micro-calcifications, which have higher surface area of hydroxyapatite than macro-calcifications [7]. Advances in PET/CT technology in the past decades have improved its spatial resolution and sensitivity while allowing for quicker scans with a lower radiation dose, accelerating the adoption of ^18^F-NaF PET/CT into clinical practice.

Approximately 10% of the population worldwide is affected by chronic kidney disease (CKD) [10]. Because impaired kidney function leads to disordered metabolism of minerals such as calcium and phosphate, arterial calcification is highly prevalent in patients with CKD [11]. Limited data is available on the application of ^18^F-NaF PET/CT to evaluate arterial calcification in CKD [7, 12]. Addressing this knowledge gap will help the development of an effective therapy to prevent or slow arterial calcification in this patient population.

To this end, we conducted a pilot study that employed a comprehensive multimodal imaging protocol to investigate the application of ^18^F-NaF PET to study arterial calcification in people with CKD. We have two study objectives. First, we aimed to determine the optimal time to perform a PET static scan after an ^18^F-NaF injection in patients with CKD. To achieve high-quality PET images, it's essential to determine the optimal time window after radiopharmaceutical administration. This timing allows the radiopharmaceutical to localize in tissues and achieve a steady state, ensuring a high signal-to-noise ratio between the target tissues, such as bone, and the reference blood pool. Converging evidence has supported acquiring a static PET image at 60 min post-injection of ^18^F-NaF for individuals without CKD [13]. It is unclear whether impaired kidney function may affect the timing for ^18^F-NaF PET imaging.

Second, we aimed to characterize and examine the relationship between arterial macro- and micro-calcification using ^18^F-NaF PET/CT in this population. While prior studies have described the spatial relationship and extent of macro- and micro-calcification in people without CKD [12, 14], these relationships have not been studied in people with CKD. In addition, since the uptake of ^18^F-NaF in aorta may be influenced by the variation of ^18^F-NaF activity in the blood pool, it is important to establish the best reference blood pool. While many studies have used right atrium as the reference blood pool [15, 16], others have also used infra-renal inferior vena cava (IVC) [17]. It is unclear whether kidney function will impact the activity of ^18^F-NaF in the blood pool. Additionally, to identify new risk factors and potential predictors of arterial calcification, we explored the relationships of aortic macro- and micro-calcification with clinical characteristics and pertinent laboratory findings in participants with CKD.

## Methods

### Participants

In this pilot cross-sectional study, seven participants with moderate CKD (stage 3b-4) and three controls without CKD were enrolled between December 2023 and June 2024. CKD patients were recruited from the Montefiore Einstein Nephrology clinics in Bronx, New York and were eligible if their age was 18 years or older and had an estimated glomerular filtration rate (eGFR) between 15 and 45 mL/min/1.73m^2^. Controls were defined as having eGFR > 60 ml/min/1.73m^2^ without prior diagnosis of CKD and had age comparable to CKD participants. Participants were excluded if they were pregnant, claustrophobic, on immunotherapy, had active hepatitis B, C or human immunodeficiency virus infection, a history of parathyroidectomy or severe bone disease (Figure S1). This study was approved by the Institutional Review Board at the Albert Einstein College of Medicine.

### Electrocardiogram-gated chest CT

To detect macro-calcification in coronary arteries, chest CT (120 kVp and 90 mA) with electrocardiogram-gating and 25 cm axial field of view (FOV) was performed. Coronary calcium CT images were reconstructed at a slice thickness of 2.5 mm. Iodinated intravenous contrast was not used because of its associated risk of contrast-induced nephropathy in people with CKD. Arterial macro-calcification was measured in CT and quantified using Aquarius iNtuition (TeraRecon) software. Macro-calcification in the coronary arteries (CAC) was quantified from the chest CT image using the standard Agatston scoring method [18], which involves multiplying the area of each calcified lesion by a density weighting factor derived from the maximum CT attenuation value measured in Hounsfield Units (HU) within that lesion. The density weighting factor is assigned according to the following thresholds: 130–199 HU = 1; 200–299 HU = 2; 300–399 HU = 3; and ≥ 400 HU = 4.^18^ The score for each lesion is the product of its area (mm^2^) and the corresponding density weighting factor, with the total Agatston score representing the sum of all individual lesion scores.

### PET/CT image acquisition and analyses

PET/CT images were acquired using the Discovery MI 20 cm 4-Ring digital PET/CT system (GE HealthCare). For structure identification and attenuation correction, low-dose, non-contrast CT scans were done with FOV of 70 cm and slice spacing of 2.79 mm and reconstructed at slice thickness of 3.75 mm. Images were analyzed using Hermes Medical Solutions (Stockholm, Sweden) workstation software. All PET images were corrected automatically for decay of the radioisotope in relation to the injection time. Standardized uptake value (SUV), the decay-corrected tissue uptake divided by the injected dose per body weight, is used as the measure for tissue uptake [19]. SUV of volume of interest (VOI) were indicated as mean (SUV_mean_) and peak (SUV_peak_, a contiguous 1cm^3^ sub-volume of high uptake).

**Fig. 1:**
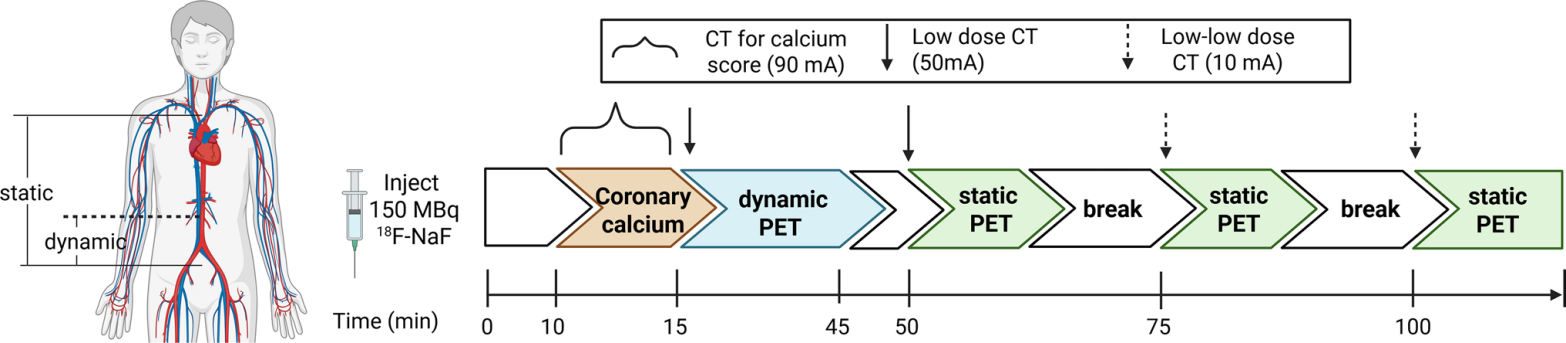
Schematics for PET/CT image acquisition. A 30-min list mode dynamic PET scan at lumbar region was performed 15 min after injection of 150 megabecquerels (MBq) ^18^F-NaF (15–45 min), followed by 3 static PET performed at 50-, 75-, and 100-min post injection [first static (1): 50–60-min, second static (2): 75–85 min, third static (3): 100–110 min]. PET images for dynamic series included 75-time frames: 40 × 15 s, 30 × 30 s, 5 × 60 s. Chest CT (120 kVp and 90 mA) with electrocardiogram-gating was done to assess macro-calcification in coronary arteries, which was quantified as Agatston score. Low dose (10–50 mA), non-contrast CT was done for structure identification and attenuation correction.

To determine the optimal time to perform a PET static scan after ^18^F-NaF injection in patients with CKD, a 30-min dynamic PET scan with 70 cm axial FOV at the lumbar region was performed 15 min after injection of 150 megabecquerels of ^18^F-NaF (Fig. 1) [20]. PET images for dynamic series were re-binned into 75-time frames: 40 × 15 s, 30 × 30 s, 5 × 60 s. Then, three 10-min static PET scans were performed from the sternal notch to aortic bifurcation at 50-, 75-, and 100-min post injection. Since bones are known to take up ^18^F-NaF, lumbar spine was used as the VOIs to determine tissue uptake of ^18^F-NaF. VOIs were drawn over a trabecular region of the L2 vertebra, avoiding the endplates and disc spaces. Since dynamic scan was only performed at the lumbar region, IVC was used to represent the blood pool for the analyses of dynamic phase. VOIs in the IVC were drawn from the L2 vertebra region to the IVC bifurcation to ensure separation from the abdominal aorta and acquired using 3–4 consecutive axial slices.

Macro- and micro-calcification of the aorta were assessed using the PET/CT images obtained at the first static scan. As previously described [21], the aorta was divided into 4 segments (Figure S2): (1) ascending aorta, slice above the aortic valve up to the first aortic branch; (2) aortic arch, next slice up to and including the slice past the last aortic branch; (3) descending thoracic aorta, next slice up to and including the diaphragm; (4) abdominal aorta, next slice up to the iliac bifurcation. The Agatston scoring method was adapted to quantify macro-calcification in the aorta obtained from low-dose CT. The parameters of low-dose CT for the aorta differ from conventional imaging protocols for obtaining Agatston score for coronary arterial calcification, which typically use a smaller FOV (~ 25 cm) and a slice thickness of 2.5 mm to optimize spatial resolution and minimize image noise [22, 23]. These differences were accounted for during image processing and interpretation to ensure consistency in scoring. For micro-calcification, the locations of the VOIs in the aortic segments were determined from PET/CT fused images and excluding suspected activity spillover from extravascular structures and artifacts within the first and last three slices of the PET image series. Since SUVs in the aorta may be influenced by variation of ^18^F-NaF activity in the blood pool, tissue-to-background ratios (TBRs) were computed as the SUVs in aorta divided by the SUV_mean_ in the blood pool, which was obtained in right atrium [8, 24]. Higher TBR indicates greater micro-calcification [7].

### Data and blood collection

Medical history was obtained via an interview and a chart review for each participant. Blood was collected after at least 8-h fasting and was measured for basic metabolic panel, cystatin C, lipid profile, markers of mineral metabolism and vascular inflammation and thrombosis. Race free eGFR was calculated using the 2021 creatinine- and cystatin C-based equation [25]. Markers of mineral metabolism included calcium, magnesium, phosphorous, vitamin D, intact parathyroid hormone (PTH), and calciprotein particles (CPP). For CPP, T_50_ or the half-maximal time of transformation from primary to secondary CPP and the size of secondary CPP aggregates were measured using nephelometry (NEPHELOstar Plus Nephelometer, BMG Labtech) and dynamic light scattering, respectively (Stunner, Unchained Lab) [26–28]. Markers of vascular inflammation and thrombosis included soluble endothelial leukocyte adhesion molecule-1 (sE-selectin), platelet endothelial cell adhesion molecule-1 (PECAM-1), penxtraxin-3, tissue factor, and thrombomodulin, and measured using Luminex MAGPIX from Millipore Sigma (cat # HCVD4MAG-67K). Samples were processed and run according to the manufacturer’s guidelines. Data was acquired on a Luminex Magpix XMAP Multiplex Reader (Luminex Technologies, RRID:SCR_023348) and analyzed using Belysa Immunoassay Curve Fitting Software using a 5-parameter logistic curve fitting method (Millipore Sigma). The rest of the blood measurements were performed in the Clinical Laboratory at Montefiore Medical Center.

### Statistical analysis

Participant characteristics were summarized for CKD and non-CKD participants and compared using Mann–Whitney U tests for continuous variables and Fisher’s exact tests for categorical variables. ^18^F-NaF uptake in the lumbar spine was assessed using SUV_mean_ plotted over time for the following PET imaging time intervals: 15–45 min (dynamic), 50–60 min (first static), 75–85 min (second static), 100–110 min (third static) for visual analyses. As described previously [13], the rate of change in uptake was computed to assess the magnitude of changes over time (i.e. % of change per minute). Paired t-tests were used to compare log-transformed SUV_mean_ values in the lumbar spine between the first and second static PET scans. For the remaining analyses, Mann–Whitney U tests were used to compare between groups, i.e. CKD vs. non-CKD, while Wilcoxon signed rank sum tests were used to compare paired data, for example comparing SUV_mean_ in right atrium and IVC among CKD patients. SUVs in aorta were obtained at the first static PET. Spearman correlation was used to assess associations between macro- and micro-calcification measures and their associations with clinical characteristics and pertinent laboratory findings. A p value < 0.05 was considered statistically significant. Due to the exploratory nature of second and third study objectives, we did not correct for multiple testing. Analyses were performed using STATA 17.0 SE (College Station, Texas).

## Results

### Optimal time to perform static PET scan among CKD patients

**Fig. 2.**
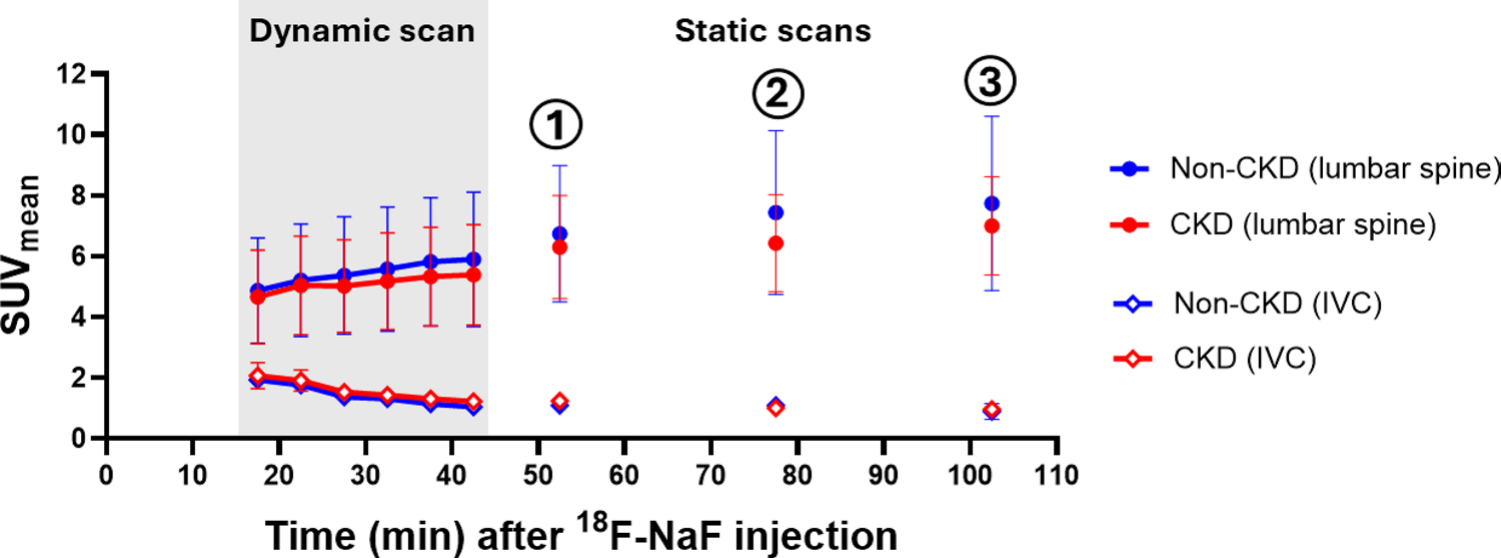
^18^F-NaF uptake in the lumbar spine and inferior vena cava (IVC) over time in participants with and without CKD. ^18^F-NaF uptake in the lumbar spine and inferior vena cava (IVC) were assessed using mean standard uptake value (SUV_mean_) plotted over time for visual analyses. Data in the dynamic scan was shown in 5-min intervals for illustration. SUV_mean_ in lumbar spine increased over time and reached a relatively steady state around the time of the first static scan, while the SUV_mean_ in IVC decreased. IVC was used to indicate blood pool because dynamic scan was performed only at the lumbar region. There was no difference in the serial uptake of ^18^F-NaF in lumbar spine between participants with and without CKD (p values range: 0.58–0.92). (1) indicates first static PET; (2) indicates second static PET; (3) indicates third static PET

Participant characteristics and laboratory values are shown in Table 1. As expected, compared to controls, CKD participants had a lower median eGFR_Cys-Cr_ (40 ml/min/1.73m^2^ (interquartile range (IQR): 31–43) vs. 95 ml/min/1.73m^2^ (IQR: 64–98); p = 0.02). Among participants with CKD (n = 7), 5 (71%) had diabetes mellitus; 2 (29%) had coronary artery disease; all had hypertension and were on statin therapy. Visual analysis of SUV_mean_ in lumbar spine over time demonstrated that ^18^F-NaF uptake in bone increased and reached a relatively steady state at approximately the time of the first static scan, which was performed ~ 50–60 min after the injection of ^18^F-NaF, while the activity in the IVC leveled off over this period (Fig. 2). Among CKD participants, the average rate of change in the lumbar spine uptake was reduced to 0.24% per minute after the first static scan, and there was no difference in the uptake between the first and second static PET scans (p = 0.47). The SUV_mean_ in the lumbar spine was not statistically different at each time point between CKD and non-CKD participants (p values range: 0.58–0.92).

**Table 1:** Participant characteristics and laboratory values by CKD status

	CKD (n = 7)	Non-CKD (n = 3)	p value
Age (year)	76 (67–77)	69 (57–70)	0.25
Women, n (%)	2 (29%)	2 (67%)	0.26
Black, n (%)	6 (86%)	1 (33%)	0.05
Diabetes mellitus, n (%)	5 (71%)	0 (0%)	0.04
Hypertension, n (%)	7 (100%)	1 (33%)	0.02
Coronary artery disease, n (%)	2 (29%)	0 (0%)	0.30
Stroke, n (%)	2 (29%)	0 (0%)	0.30
Aspirin use, n (%)	3 (43%)	0 (0%)	0.17
Statin use, n (%)	7 (100%)	1 (33%)	0.02
Serum creatinine (mg/dL)	1.79 (1.32.22)	0.92 (0.62–1.01)	0.02
Serum cystatin C (mg/L)	1.65 (1.47–2.10)	0.88 (0.84–1.10)	0.02
eGFRcys-Cr (ml/min/1.73m^2^)	40 (31–43)	95 (64–98)	0.02
Serum calcium (mg/dL)	9.4 (9.2–9.6)	9.4 (8.9–9.5)	0.91
Serum phosphorous (mg/dL)	3.4 (3.2–3.6)	3.3 (2.6–3.8)	0.72
Serum magnesium (mg/dL)	1.8 (1.7–2.3)	2.1 (1.9–2.1)	0.83
Parathyroid hormone (pg/mL)	104 (70–125)	93 (74–110)	0.83
25-vitamin D (ng/ml)	39 (30–42)	52 (16–77)	0.48
T_50_ (minute)	199 (164–223)	227 (129–244)	0.52
Secondary CPP size (nm)	188 (151–239)	206 (179–278)	0.38
Albumin (g/dL)	4.2 (4.0–4.3)	4.2 (4.0–4.2)	0.87
Total cholesterol (mg/dL)	140 (104–170)	193 (174–196)	0.03
High-density lipoprotein (mg/dL)	41 (31–48)	57 (46–58)	0.17
Low-density lipoprotein (mg/dL)	71 (58–86)	119 (113–126)	0.02
sE-selectin (ng/mL)	97 (45–135)	n/a	n/a
PECAM-1 (ng/mL)	1.3 (1.1–1.9)	n/a	n/a
Pentraxin-3 (pg/mL)	450 (217–597)	n/a	n/a
Tissue factor (pg/mL)	204 (168–222)	n/a	n/a
Thrombomodulin (ng/mL)	7.7 (5.9–10.3)	n/a	n/a

*eGFR*_*cys-Cr*_ estimated glomerular filtration rate estimated using serum creatinine and cystatin C, *T50* time for half-maximal transformation from priary to secondary calciprotein particles (CPP), *sE-selectin* soluble endothelial leukocyte adhesion molecule-1, *PECAM-1* platelet endothelial cell adhesion molecule-1

Median (Interquartile range, IQR) was listed for continuous variables and frequency (percentage) was listed for categorical variables. Mann–Whitney U tests were used to compare continuous variables and Pearson’s chi-squared tests, or Fisher’s exact tests were used to compare categorical variables between CKD and non-CKD groups

### SUV_mean_ in right atrium and IVC

**Fig. 3:**
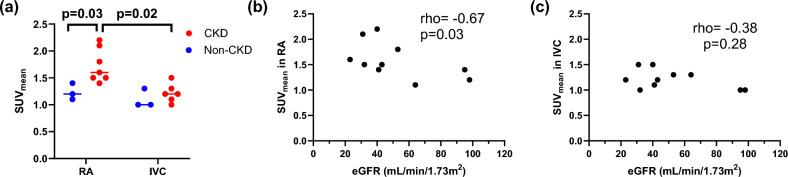
SUV_mean_ in right atrium (RA) and inferior vena cava (IVC). **a** SUV_mean_ in RA was higher in participants with CKD than non-CKD controls, but there was no difference in SUV_mean_ in IVC. Among participants with CKD, SUV_mean_ in right atrium is higher than in IVC. **b** SUV_mean_ in RA was negatively correlated with eGFR, while **c** there was no correlation between SUV_mean_ in IVC and eGFR

At the first static PET, SUV_mean_ in right atrium was higher in participants with CKD than non-CKD controls (Median (IQR): 1.6 (1.5–2.1) vs. 1.2 (1.1–1.4), p = 0.03), while there was no difference in SUV_mean_ in IVC (p = 0.31; Fig. 3a). Among participants with CKD, SUV_mean_ in right atrium was higher than in IVC (p = 0.02). Lower eGFR was correlated with higher SUV_mean_ in the right atrium (rho = − 0.67, p = 0.03, Fig. 3b) but not with SUV_mean_ in IVC (p = 0.28; Fig. 3c).

### Arterial macro- and micro-calcification spatial patterns

**Fig. 4:**
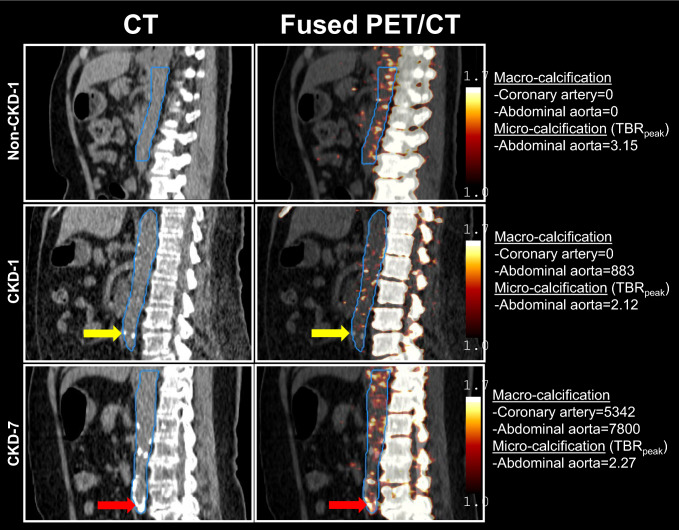
Representative sagittal images of macro- and micro-calcification in abdominal aorta from 1 participant without CKD and 2 participants with CKD. VOIs for abdominal aorta are outlined in blue. Low dose CT imaging shows macro-calcification indicated by radiopacity. Fused PET/CT imaging shows micro-calcification indicated by high ^18^F-NaF uptake and represented by peak tissue-to-background ratio (TBR_peak_). Yellow arrows indicate lesions with macro-calcification on CT without any ^18^F-NaF uptake on PET. Red arrows indicate colocalized lesions of macro-calcification and ^18^F-NaF uptake. Color scale indicates the degree of radioactivity in TBR_peak_

CAC score, macro- and micro-calcification in aorta for each participant are listed in Table 2***.*** Representative images from 2 participants with CKD and 1 non-CKD control are shown in Fig. 4 to illustrate the spatial distribution and co-localization between macro- and micro-calcification in the abdominal aorta. Participant non-CKD-1 had a CAC score of 0; in abdominal aorta, the calcium score is 0 while micro-calcification was present as indicated by the high TBR_peak_ or hot spots. Participant CKD-1 also had a CAC score of 0; in abdominal aorta, spots of focal macro-calcification and micro-calcification both existed but did not overlap. Participant CKD-7 had macro-calcification in both the coronary arteries and abdominal aorta as well as micro-calcification demonstrated as hot spots throughout the abdominal segment with some of them overlapping spatially with macro-calcification.

**Table 2:** Arterial macro-calcification and micro-calcification in participants with CKD (n = 7) and non-CKD controls (n = 3)

	Macro-Calcification (CT; Agatston score)						Micro-calcification (PET; TBR_peak_)			
	Coronary artery	Ascending aorta	Aortic arch	Descending thoracic aorta	Abdominal aorta	Total aorta	Ascending aorta	Aortic arch	Descending thoracic aorta	Abdominal aorta
CKD										
1	0	330	195	0	883	1408	1.31	1.57	3.59	2.12
2	30	0	71	0	153	225	1.44	2.02	2.73	2.66
3	305	1057	2265	440	5193	8955	1.31	1.24	1.40	1.40
4	473	0	646	0	482	1128	1.39	2.31	2.03	1.46
5	482	0	0	184	1337	1521	1.71	2.73	2.80	2.41
6	2313	0	0	391	6436	6828	1.41	1.62	2.39	1.60
7	5342	511	2397	2883	7800	13,591	1.31	1.42	1.57	2.27
Non-CKD										
1	0	0	0	0	0	0	1.57	3.75	4.44	3.15
2	0	0	0	0	0	0	1.42	1.69	1.94	1.96
3	559	0	0	215	1793	2009	1.29	2.52	2.25	1.55

### Arterial macro- and micro-calcification among participants with CKD

**Fig. 5:**
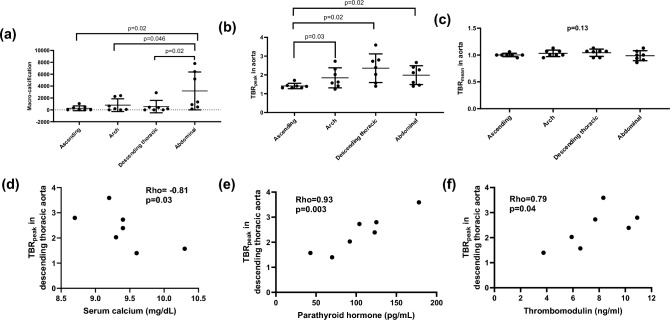
Aortic macro-calcification and micro-calcification among CKD participants (n = 7). Among the 4 aortic segments, **a** abdominal aorta had the greatest macro-calcification on CT, whereas **b** the ascending aorta had the lowest micro-calcification quantified by TBR_peak_. **c** There was no difference in TBR_mean_ among different aortic segments. **d**–**f** TBR_peak_ in the descending thoracic aorta was negatively correlated with serum calcium and negatively correlated with parathyroid hormone and thrombomodulin levels

Among participants with CKD, 6 had a CAC score > 0. CAC was not correlated with aortic macro- or micro-calcification (Table S1). The correlation between CAC and macro-calcification in the abdominal aorta was approaching significance with a p-value of 0.052 (rho = 0.75). Among the 4 aortic segments, abdominal aorta had the greatest macro-calcification, and ascending aorta had the lowest TBR_peak_, while there was no difference in TBR_mean_ (Fig. 5a–c, Figure S3). Macro-calcification in the descending thoracic aorta was correlated with macro-calcification in the abdominal aorta (rho = 0.93, p = 0.003; Table S1). Macro-calcification in the ascending aorta had a negative correlation with TBR_peak_ in that segment (rho = − 0.86, p = 0.01), while there were no correlations between macro-calcification and TBR_peak_ in other aortic segments. There was no correlation between macro-calcification and TBR_mean_ within each aortic segment (Table S2).

### Factors associated with macro- and micro-calcification in participants with CKD

CKD participants with greater macro-calcification in the descending thoracic and abdominal aorta were older (Table S3). Having coronary artery disease was associated with greater CAC and macro-calcification in the abdominal aorta (rho = 0.79, p = 0.03 for both). Having diabetes was associated with greater TBR_peak_ in the ascending aorta (rho = 0.79, p = 0.03), while history of stroke was associated with greater TBR_peak_ in the aortic arch (rho = 0.79, p = 0.03). For the laboratory values, low-density lipoprotein level negatively correlated with macro-calcification in the coronary arteries and descending thoracic and abdominal aorta (rho ranged from − 0.79 to − 0.89). Serum magnesium was negatively correlated with macro-calcification in both ascending aorta and arch (rho = − 0.85, p = 0.01; and rho = − 0.92, rho = 0.004, respectively) but positively correlated with TBR_peak_ in these 2 aorta segments (rho = 0.84, p = 0.02 for both). TBR_peak_ in the descending thoracic aorta was negatively correlated with serum calcium (rho = − 0.81, p = 0.03), and positively correlated with PTH (rho = 0.93, p = 0.003) and thrombomodulin levels (rho = 0.79, p = 0.04; Fig. 5d–f, Table S3).

## Discussion

In this pilot study, we determined the optimal time to perform PET scan after ^18^F-NaF injection in people living with CKD, successfully characterized and compared aortic macro- and micro-calcification using the latest PET technology, and examined their associated clinical and laboratory factors. These findings may inform the development of ^18^F-NaF PET/CT imaging and analysis protocols for patients with CKD, who have a high prevalence of arterial calcification.

For the first time, we report the time course of bone uptake and blood pool activity of ^18^F-NaF in people with CKD, allowing us to determine an appropriate time for static measurement of these parameters. In people without kidney disease, the optimal time to perform PET imaging after injection of ^18^F-NaF is approximately 60 min [13]. In our study, we found no difference in the time to reach the optimal uptake in the lumbar spine between participants with and without CKD. Similar to what was reported by Kurdziel et al. [13], we found that 50–60 min after the injection of ^18^F-NaF, the rate of increase in bone uptake became very low while the activity in IVC stabilized during this period indicating a high signal-to-noise ratio. There was no difference in the radiopharmaceutical uptake in bone between ~ 50 and 75 min post-injection. These findings are very similar to that of another ^18^F radiopharmaceutical—[^18^F]-Fluoro-2-deoxy-2-D-glucose (^18^F-FDG), for which there was no significant difference in the serial tissue uptake of FDG for up to 3 h among patients with different degrees of kidney dysfunction [29]. Based on these findings, we recommend that PET imaging can be also performed ~ 60 min after ^18^F-NaF injection for optimal image analyses in people with moderate CKD, aligning with that of people without CKD.

Our findings indicate that SUV of ^18^F-NaF in the right atrium 50–60 min after the injection was much higher than that in the infra-renal IVC in participants with CKD, while there was no difference in non-CKD controls. SUVs in the aorta may be influenced by the variation of ^18^F-NaF activity in the blood pool; therefore, it is important to establish the right reference blood pool for the calculation of TBR. As stated earlier, prior studies have used either right atrium or infra-renal IVC as reference blood pool [15–17]. In the abdomen, since the aorta and IVC run parallel and close to each other, to obtain VOIs of the IVC, separate from aorta, the IVC was drawn infra-renally in our study. We found a significant inverse relationship between kidney function and SUV_mean_ in the right atrium, whereas no such correlation was observed in IVC. Furthermore, participants with CKD had markedly higher SUV_mean_ in the right atrium compared to the IVC. The etiology of this differential blood activity remains unclear. While the elevated ^18^F-NaF activity in the right atrium may be attributable to imaging artifact, we propose a physiological explanation based on the distinct venous inflow patterns. Unlike the infra-renal IVC, the right atrium receives venous return from the superior vena cava, coronary sinus, hepatic circulation, and renal veins. In CKD, reduced renal clearance of ^18^F-NaF likely results in prolonged systemic circulation of the tracer, potentially increasing its activity in the right atrium. Our findings suggest that the right atrium may be a more appropriate region to be used as a reference blood pool for the calculation of TBR than infra-renal IVC in people with CKD.

We successfully characterized and compared macro- and micro-calcification in participants with CKD. Prior studies showed that macro-calcification in coronary arteries correlates with that of the aorta [30]. However, this relationship was not observed in our study. This is likely due to small sample size (n = 7). Note that the correlation between CAC and abdominal aorta in our study was approaching a statistical significance (p = 0.052). Additionally, aorta has a different susceptibility to calcification than the coronary arteries [31, 32]. Consistent with prior findings [12, 33], we found that abdominal aorta has greater macro-calcification than other aortic segments. The differences in aortic segments may be explained by their anatomic and embryonic differences since vascular smooth muscle cells involved in calcification have distinct origins in development: those that form the wall of the ascending and descending thoracic aorta originate from the neural crest, whereas those in the abdominal aorta arise from mesoderm [34].

We did not observe correlations between macro-calcification by CT and TBR_peak_ of ^18^F-NaF within each aortic segment except in the ascending aorta, in which macro-calcification was negatively correlated with TBR_peak_. Derlin et al., found that in a cohort of 45 participants, there was a significant correlation between ^18^F-NaF uptake and macro-calcification score (p < 0.001), but the correlation was relatively weak with a rho of 0.36 [14]. It is unclear whether our findings are due to small sample size or reflect the unique pathogenesis of arterial calcification in CKD. In patients with CKD, arterial macro-calcification is more likely to occur in the arterial media, but micro-calcification is more likely to be detected in the intima [35]. Similar to prior studies, we identified macro-calcified lesions with and without ^18^F-NaF uptake. For example, Derlin et al. [12], reported that only 20% of macro-calcified lesions showed an increased uptake in ^18^F-NaF. Uptake in ^18^F-NaF in these lesions likely represents growing lesion of calcification, whereas the one without uptake might represent stable disease. These findings underscore the value of characterizing both macro- and micro-calcification and imply that early intervention, prior to the onset of macro-calcification, may be beneficial.

Several interesting observations were made while exploring the relationship between clinical factors and arterial calcification. Consistent with prior literature [36], greater macro-calcification was correlated with older age and the history of coronary artery disease. Macro-calcification was negatively correlated with cholesterol levels, and this may have been confounded by the different doses and duration of statin therapy. In the descending thoracic aorta, greater TBR_peak_ was negatively correlated with serum calcium but positively correlated with PTH level. Patients with impaired kidney function have abnormal metabolism of minerals such as calcium and phosphate [37]. They may develop hypocalcemia due to decreased renal production of active vitamin D, which helps gastrointestinal absorption of calcium. Hypocalcemia then stimulates the production of PTH, which induces bone turnover to increase serum calcium and contribute to arterial calcification. In rats with impaired kidney function, PTH replacement induced high bone turnover and arterial calcification [38] and that lowering of PTH using cinacalcet inhibited calcification [39]. In the descending thoracic aorta, we also found that the higher thrombomodulin level was correlated with greater TBR_peak_. Thrombomodulin, a glycoprotein on the surface of endothelial cells, is involved in crucial biological processes such as coagulation, innate immunity, and inflammation [40]. In vasculature, thrombomodulin prevents thrombosis and inflammation [41], and may protect against phosphate induced calcification of vascular smooth muscle cells, which are the key cell type in the pathogenesis of arterial calcification [42]. To our knowledge, no prior studies have examined the relationship between thrombomodulin and ^18^F-NaF uptake in the aorta.

Our pilot study has several limitations. This study included only three non-CKD participants as controls, which limits the robustness of comparisons between CKD participants (n = 7) and non-CKD controls. Given the small sample size, our study may have been underpowered to detect certain differences. Additionally, we were unable to adjust for potential confounders, such as statin use. The cross-sectional design does not allow us to infer causality. Including individuals with milder CKD (i.e., stages 1–3a) in a longitudinal design may enable evaluation of the progression of micro- and macro-calcification as kidney function declines. Although prior studies have utilized ^1^⁸F-NaF PET to evaluate peripheral arterial disease [43, 44], we did not include this assessment in our pilot study, as it was beyond the scope of our investigation and PET/CT imaging was performed up to the bifurcation of the aorta. We also did not assess ^18^F-NaF uptake in the coronary arteries because it requires intravenous administration of iodinated contrast and patients with CKD have a high risk of developing contrast-induced nephropathy. Lastly, given the exploratory nature of our second and third study objectives, we did not apply corrections for multiple testing. As a result, these findings should be considered hypothesis-generating rather than conclusive and should be interpreted with appropriate caution.

Despite limitations, our study has multiple strengths. Our findings help define the optimal timing of ^18^F-NaF PET imaging using a 30-min dynamic scan and 3 static scans balancing data acquisition and participant comfort over almost 2 h. We showed that the right atrium is a more appropriate reference blood pool to calculate TBR in the aorta than IVC in people with CKD. Not only did we successfully characterize arterial calcification using ^18^F-NaF PET/CT in people with CKD, but we also conducted a comprehensive assessment in each participant incorporating dynamic scanning, detailed characterization of arterial macro- and micro-calcification, and thorough measurements of serum markers for mineral metabolism and vascular inflammation and thrombosis. Lastly, as arterial calcification takes years to develop and its prevalence increases as kidney function declines [31, 45], by studying patients with CKD before they reach end stage kidney disease and need dialysis, it allows identification of those at a high risk of developing arterial calcification while providing a sufficient window of time for successful intervention [31, 45]. In summary, our findings help to establish important technical specifications in image acquisition and analyses of ^18^F-NaF PET using the latest technology, provide a foundation for its longitudinal investigations, and support the potential application of ^18^F-NaF PET imaging in the management of arterial calcification in CKD.

## New knowledge gained

^18^F-NaF PET detects arterial micro-calcification, allowing for early detection and intervention. There is limited data on its use in people with CKD, who have a high prevalence of arterial calcification. We found that the optimal time to perform PET imaging after the injection of ^18^F-NaF is ~ 60 min in patients with CKD, aligning with that of people without CKD. We also found that the right atrium is more appropriate to be used as a reference blood pool than IVC for people with CKD. In addition, our study revealed novel links between ^18^F-NaF uptake in aorta and levels of serum parathyroid hormone and thrombomodulin. Our findings help to establish important technical specifications in image acquisition and analyses of ^18^F-NaF PET using the latest technology and provide a foundation for future longitudinal investigations.

## Supplementary Information

Below is the link to the electronic supplementary material.

Supplementary Material 1

## Data Availability

Individual level data are displayed in the main manuscript and the supplementary materials when appropriate. The raw and de-identified data are available from the corresponding author on reasonable request.

## Abbreviations

CPP: Calciprotein particle
CKD: Chronic kidney disease
CT: Computed tomography
CAC: Coronary artery macro-calcification
eGFR: Estimated glomerular filtration rate
FOV: Field of view
^18^F-NaF: ^18^Fluorine-sodium fluoride
IVC: Inferior Vena Cava
PTH: Parathyroid hormone
PECAM-1: Platelet endothelial cell adhesion molecule-1
PET: Positron emission tomography
RA: Right atrium
SUV: Standard uptake value
sE-selectin: Soluble endothelial leukocyte adhesion molecule-1
TBR: Tissue-to-background ratio
VOI: Volume of interest
