# Supplementary material for: 18Fluorine-sodium fluoride positron emission tomography to evaluate arterial calcification in patients with chronic kidney disease: a pilot study
Source: Int J Cardiovasc Imaging. 2025 Nov 13;41(12):2415–26. doi: 10.1007/s10554-025-03553-0 (PMC12628283; doi:10.1007/s10554-025-03553-0)
Supplement: Supplementary file 1 — Supplementary Material 1 [file 10554_2025_3553_MOESM1_ESM.docx]

**SUPPLEMENTARY MATERIALS**

**Title:** ^18^Fluorine-Sodium Fluoride Positron Emission Tomography to Evaluate Arterial Calcification in Patients with Chronic Kidney Disease: A Pilot Study

**Authors**: Aspan M. Shokrekhuda,*^1^ Rylen Stratford,*^1^ Wenzhu Mowrey,^2^ Na Song,^1^ Kexin Zhang,^3^ Sara Saini,^3^ Mario T. Di Dea,^1^ Gabriel Duah,^1^ Zahra Karimi,^1^ Nicholas E. S. Sibinga,^4,5,6^ Yonatan Schwartz,^7^ Jeffrey M. Levsky,^4,7^ Marc R. Dweck,^8^ Renee Moadel,^1^ Lionel S. Zuckier,^9^ Mark I. Travin,^1,4^ Wei Chen^3,5,6,10^

**Supplementary Figures**

**Figure S1. Participant flow chart**


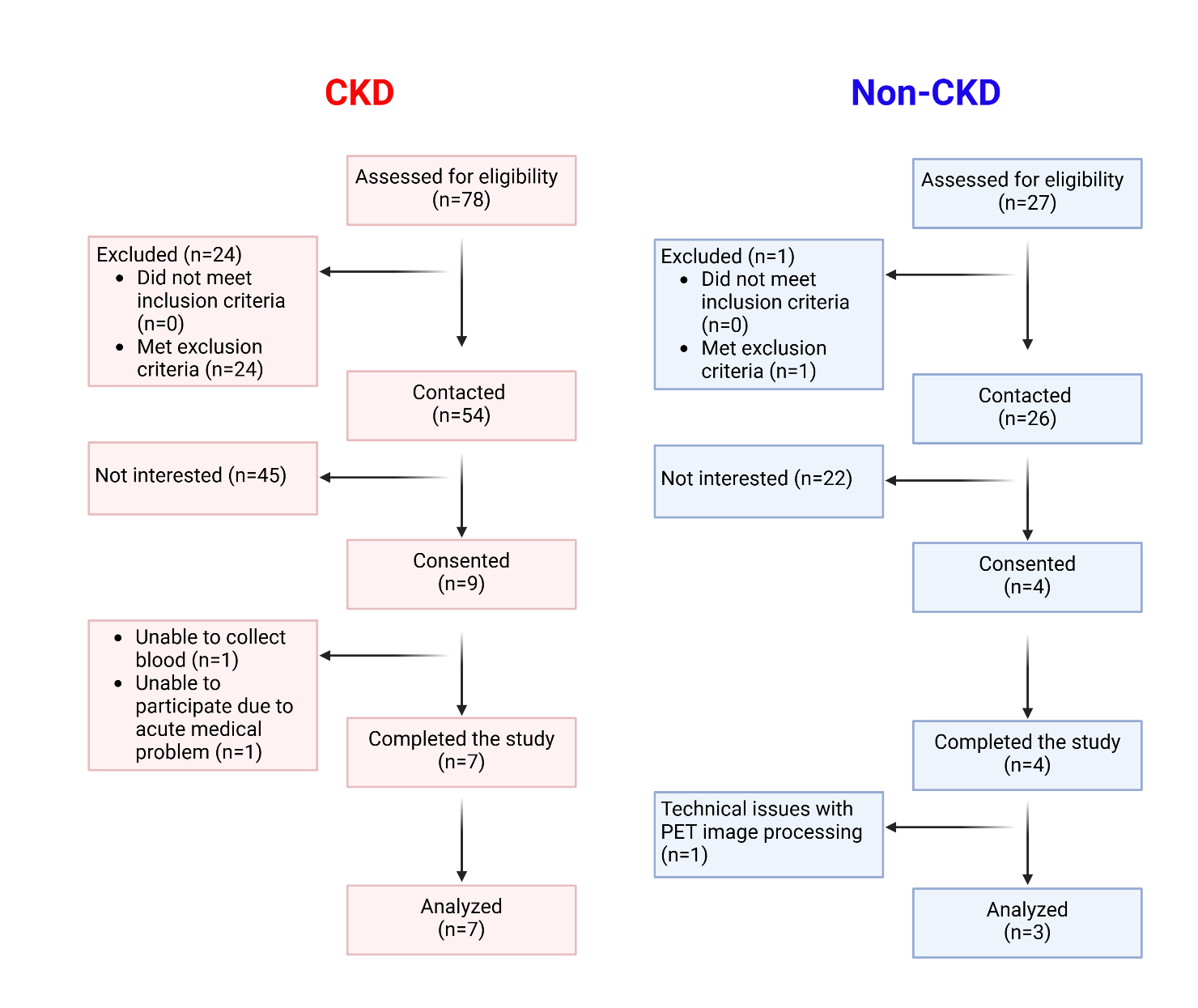


**Figure S2. Volume of interest (VOIs) in aorta and reference blood pool.** Aorta was divided into ascending, arch, descending thoracic, and abdominal segments. SUV_mean_ in the blood pool was obtained from right atrium and inferior vena cava (IVC).


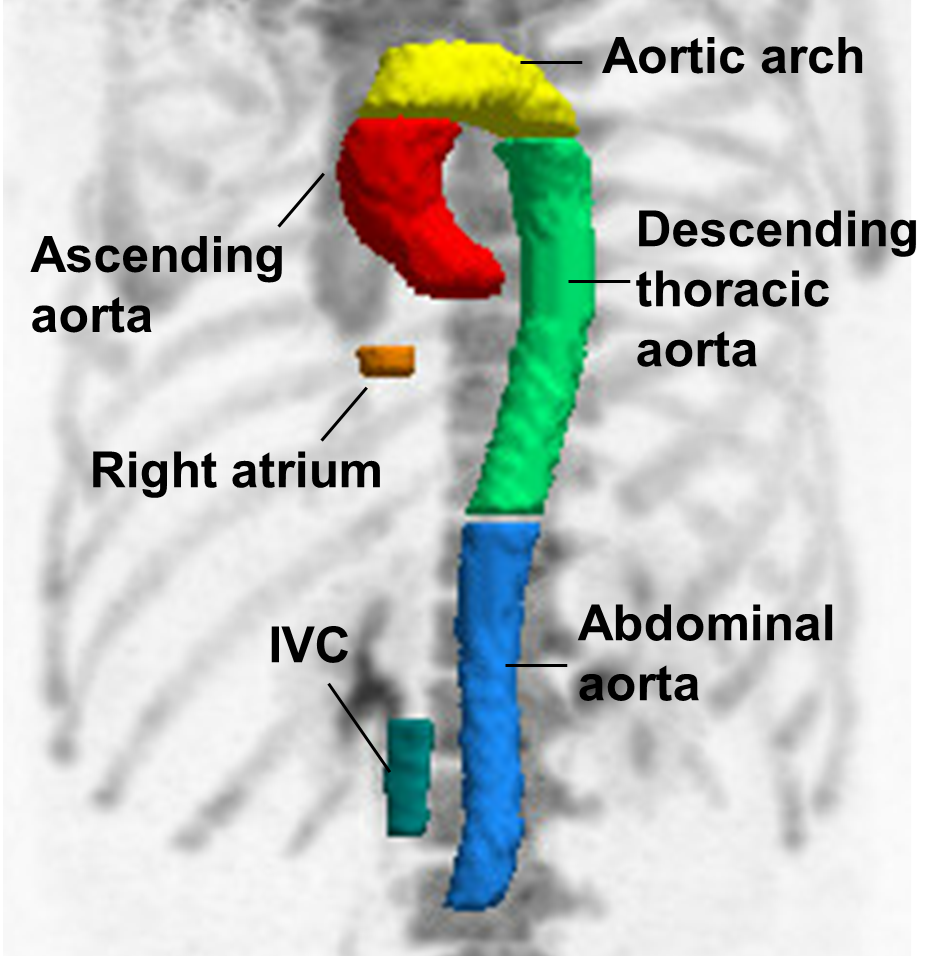


**Figure S3. Macro-calcification normalized by volume of interest (VOI; mL) in different aortic segments**


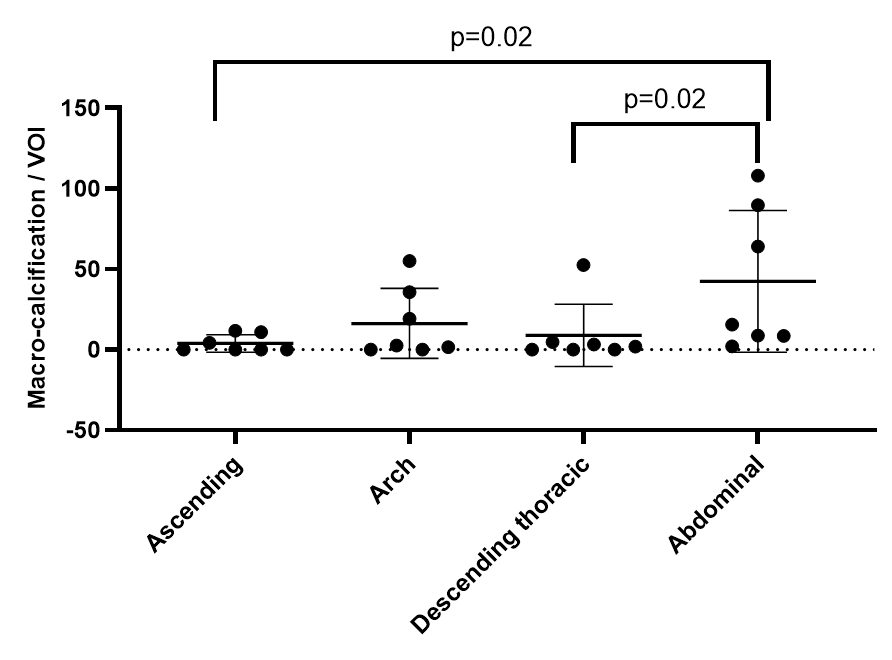


**Supplementary Tables**

**Table S1. Correlation matrix of arterial macro-calcification and micro-calcification (in TBR_peak_) among participants with CKD (n=7).** Spearman correlation was used, and correlations with p<0.05 are highlighted in gray.

|  |  |  | Macro-calcification (CT) | | | | | | Micro-calcification (PET, TBRpeak) | | | |
| --- | --- | --- | --- | --- | --- | --- | --- | --- | --- | --- | --- | --- |
|  |  |  | CAC | Ascending aorta | Aortic arch | Descending thoracic aorta | Abdominal aorta | Total aorta | Ascending aorta | Aortic arch | Descending thoracic aorta | Abdominal aorta |
| Macro-calcification (CT) | CAC | Rho | 1.00 |  |  |  |  |  |  |  |  |  |
|  |  | p | n/a |  |  |  |  |  |  |  |  |  |
|  | Ascending aorta | Rho | -0.06 | 1.00 |  |  |  |  |  |  |  |  |
|  |  | p | 0.90 | n/a |  |  |  |  |  |  |  |  |
|  | Aortic arch | Rho | 0.05 | 0.78 | 1.00 |  |  |  |  |  |  |  |
|  |  | p | 0.91 | 0.04 | n/a |  |  |  |  |  |  |  |
|  | Descending thoracic aorta | Rho | 0.70 | 0.59 | 0.39 | 1.00 |  |  |  |  |  |  |
|  |  | p | 0.08 | 0.16 | 0.38 | *n/a* |  |  |  |  |  |  |
|  | Abdominal aorta | Rho | 0.75 | 0.49 | 0.25 | 0.93 | 1.00 |  |  |  |  |  |
|  |  | p | 0.052 | 0.26 | 0.59 | 0.003 | *n/a* |  |  |  |  |  |
|  | Total aorta | Rho | 0.64 | 0.67 | 0.41 | 0.96 | 0.96 | 1.00 |  |  |  |  |
|  |  | p | 0.12 | 0.10 | 0.36 | 0.001 | 0.001 | *n/a* |  |  |  |  |
| Micro-calcification (PET, TBR_peak_) | Ascending aorta | Rho | 0.11 | -0.86 | -0.82 | -0.35 | -0.37 | -0.48 | 1.00 |  |  |  |
|  |  | p | 0.81 | 0.01 | 0.02 | 0.45 | 0.41 | 0.27 | *n/a* |  |  |  |
|  | Aortic arch | Rho | 0.04 | -0.91 | -0.67 | -0.59 | -0.54 | -0.64 | 0.85 | 1.00 |  |  |
|  |  | p | 0.94 | 0.005 | 0.10 | 0.16 | 0.22 | 0.12 | 0.01 | *n/a* |  |  |
|  | Descending thoracic aorta | Rho | -0.43 | -0.51 | -0.68 | -0.67 | -0.46 | -0.57 | 0.44 | 0.54 | 1.00 |  |
|  |  | p | 0.34 | 0.24 | 0.09 | 0.10 | 0.29 | 0.18 | 0.32 | 0.22 | *n/a* |  |
|  | Abdominal aorta | Rho | 0.00 | -0.39 | -0.38 | -0.22 | -0.25 | -0.32 | 0.56 | 0.43 | 0.57 | 1.00 |
|  |  | p | 1.00 | 0.38 | 0.40 | 0.63 | 0.59 | 0.48 | 0.20 | 0.34 | 0.18 | *n/a* |

Abbreviations: CAC, coronary artery calcification.

**Table S2. Correlation matrix of arterial macro-calcification and micro-calcification (in TBR_mean_) among participants with CKD (n=7).** Spearman correlation was used, and correlations with p<0.05 are highlighted in gray.

|  |  |  | Macro-calcification (CT) | | | | | | Micro-calcification (PET, TBR_mean_) | | | |
| --- | --- | --- | --- | --- | --- | --- | --- | --- | --- | --- | --- | --- |
|  |  |  | CAC | Ascending aorta | Aortic arch | Descending thoracic aorta | Abdominal aorta | Total aorta | Ascending aorta | Aortic arch | Descending thoracic aorta | Abdominal aorta |
| Micro-calcification (PET, TBR_mean_) | Ascending aorta | Rho | 0.13 | -0.37 | -0.13 | 0.00 | -0.27 | -0.27 | 1.00 |  |  |  |
|  |  | p | 0.78 | 0.42 | 0.77 | 1.00 | 0.56 | 0.56 | n/a |  |  |  |
|  | Aortic arch | Rho | 0.15 | -0.44 | -0.25 | -0.17 | -0.24 | -0.32 | 0.56 | 1.00 |  |  |
|  |  | p | 0.75 | 0.32 | 0.58 | 0.71 | 0.60 | 0.49 | 0.19 | n/a |  |  |
|  | Descending thoracic aorta | Rho | 0.37 | -0.58 | -0.42 | 0.00 | -0.07 | -0.21 | 0.77 | 0.86 | 1.00 |  |
|  |  | p | 0.41 | 0.17 | 0.35 | 1.00 | 0.87 | 0.66 | 0.04 | 0.01 | n/a |  |
|  | Abdominal aorta | Rho | 0.72 | -0.32 | -0.17 | 0.32 | 0.34 | 0.22 | 0.34 | 0.77 | 0.77 | 1.00 |
|  |  | p | 0.07 | 0.49 | 0.71 | 0.49 | 0.45 | 0.64 | 0.46 | 0.04 | 0.04 | n/a |

Abbreviations: CAC, coronary artery calcification.

**Table S3. Correlations of clinical characteristics and laboratory values with arterial macro- and micro-calcification (in TBR_peak_) among participants with CKD (n=7).** Spearman correlation was used, and correlations with p<0.05 are highlighted in gray.

|  |  | **Macro-calcification (CT)** | | | | | | **Micro-calcification (PET, TBR_peak_)** | | | |
| --- | --- | --- | --- | --- | --- | --- | --- | --- | --- | --- | --- |
|  |  | **CAC** | **Ascending aorta** | **Aortic arch** | **Descending thoracic aorta** | **Abdominal aorta** | **Total aorta** | **Ascending aorta** | **Aortic arch** | **Descending thoracic aorta** | **Abdominal aorta** |
| Age | Rho | 0.63 | 0.59 | 0.59 | 0.80 | 0.85 | 0.85 | -0.49 | -0.67 | -0.74 | -0.58 |
|  | p | 0.13 | 0.17 | 0.16 | 0.03 | 0.02 | 0.02 | 0.27 | 0.10 | 0.06 | 0.18 |
| Women (Y/N) | Rho | 0.47 | 0.09 | 0.64 | 0.16 | 0.16 | 0.16 | -0.16 | 0.00 | -0.47 | -0.16 |
|  | p | 0.28 | 0.85 | 0.12 | 0.73 | 0.73 | 0.73 | 0.73 | 1.00 | 0.28 | 0.73 |
| Black (Y/N) | Rho | -0.61 | -0.45 | -0.62 | -0.64 | -0.61 | -0.61 | 0.20 | 0.41 | 0.41 | -0.20 |
|  | p | 0.14 | 0.31 | 0.14 | 0.13 | 0.14 | 0.14 | 0.66 | 0.36 | 0.36 | 0.66 |
| Diabetes mellitus (Y/N) | Rho | 0.63 | -0.70 | -0.32 | 0.00 | 0.00 | -0.16 | 0.79 | 0.63 | 0.00 | 0.47 |
|  | p | 0.13 | 0.08 | 0.49 | 1.00 | 1.00 | 0.73 | 0.03 | 0.13 | 1.00 | 0.28 |
| Coronary artery disease (Y/N) | Rho | 0.79 | 0.09 | 0.08 | 0.66 | 0.79 | 0.63 | 0.00 | -0.32 | -0.32 | 0.00 |
|  | p | 0.03 | 0.85 | 0.87 | 0.11 | 0.03 | 0.13 | 1.00 | 0.49 | 0.49 | 1.00 |
| Stroke (Y/N) | Rho | 0.16 | -0.52 | -0.24 | -0.33 | -0.32 | -0.32 | 0.47 | 0.79 | 0.16 | 0.00 |
|  | p | 0.73 | 0.23 | 0.61 | 0.47 | 0.49 | 0.49 | 0.28 | 0.03 | 0.73 | 1.00 |
| Aspirin use (Y/N) | Rho | 0.43 | -0.72 | -0.58 | -0.15 | 0.00 | -0.14 | 0.58 | 0.72 | 0.14 | -0.14 |
|  | p | 0.33 | 0.07 | 0.17 | 0.75 | 1.00 | 0.76 | 0.17 | 0.07 | 0.76 | 0.76 |
| eGFR_cys-Cr_ | Rho | 0.36 | -0.49 | -0.31 | -0.19 | 0.07 | -0.07 | 0.14 | 0.50 | 0.14 | -0.36 |
|  | p | 0.43 | 0.26 | 0.50 | 0.69 | 0.88 | 0.88 | 0.76 | 0.25 | 0.76 | 0.43 |
| Total cholesterol | Rho | -0.86 | 0.02 | 0.11 | -0.70 | -0.68 | -0.64 | -0.46 | -0.14 | 0.29 | -0.11 |
|  | p | 0.01 | 0.97 | 0.82 | 0.08 | 0.09 | 0.12 | 0.29 | 0.76 | 0.53 | 0.82 |
| High-density lipoprotein | Rho | -0.90 | 0.38 | 0.29 | -0.49 | -0.56 | -0.38 | -0.58 | -0.23 | 0.18 | -0.23 |
|  | p | 0.01 | 0.40 | 0.53 | 0.27 | 0.19 | 0.40 | 0.18 | 0.61 | 0.70 | 0.61 |
| Low-density lipoprotein | Rho | -0.79 | -0.32 | -0.09 | -0.82 | -0.89 | -0.86 | -0.04 | 0.21 | 0.21 | -0.04 |
|  | p | 0.04 | 0.49 | 0.85 | 0.03 | 0.01 | 0.01 | 0.94 | 0.64 | 0.64 | 0.94 |
| Serum calcium | Rho | 0.36 | 0.58 | 0.65 | 0.69 | 0.52 | 0.58 | -0.31 | -0.74 | -0.81 | -0.22 |
|  | p | 0.43 | 0.18 | 0.12 | 0.09 | 0.23 | 0.18 | 0.50 | 0.06 | 0.03 | 0.64 |
| Serum phosphorous | Rho | 0.00 | 0.06 | -0.13 | 0.24 | 0.14 | 0.09 | 0.13 | -0.38 | -0.13 | 0.29 |
|  | p | 1.00 | 0.90 | 0.79 | 0.60 | 0.76 | 0.85 | 0.79 | 0.40 | 0.79 | 0.53 |
|  |  |  |  |  |  |  |  |  |  |  |  |
| **Table S3. [continued]** | | | | | | | | | | | |
|  |  | **Macro-calcification (CT)** | | | | | | **Micro-calcification (PET, TBR_peak_)** | | | |
|  |  | **CAC** | **Ascending aorta** | **Aortic arch** | **Descending thoracic aorta** | **Abdominal aorta** | **Total aorta** | **Ascending aorta** | **Aortic arch** | **Descending thoracic aorta** | **Abdominal aorta** |
| Serum magnesium | Rho | -0.09 | -0.85 | -0.92 | -0.51 | -0.45 | -0.58 | 0.84 | 0.84 | 0.71 | 0.62 |
|  | p | 0.85 | 0.01 | 0.004 | 0.24 | 0.31 | 0.17 | 0.02 | 0.02 | 0.07 | 0.14 |
| Parathyroid hormone | Rho | -0.43 | -0.47 | -0.77 | -0.59 | -0.36 | -0.46 | 0.18 | 0.46 | 0.93 | 0.29 |
|  | p | 0.34 | 0.28 | 0.04 | 0.16 | 0.43 | 0.29 | 0.70 | 0.29 | 0.003 | 0.53 |
| 25-vitamin D | Rho | 0.11 | -0.06 | -0.16 | 0.21 | 0.11 | 0.14 | 0.14 | -0.02 | -0.49 | -0.67 |
|  | p | 0.82 | 0.90 | 0.73 | 0.66 | 0.82 | 0.76 | 0.76 | 0.97 | 0.27 | 0.10 |
| T_50_ | Rho | -0.36 | -0.08 | 0.02 | -0.22 | -0.46 | -0.32 | 0.14 | 0.14 | -0.36 | -0.43 |
|  | p | 0.43 | 0.87 | 0.97 | 0.63 | 0.29 | 0.48 | 0.76 | 0.76 | 0.43 | 0.34 |
| Secondary CPP size | Rho | 0.43 | -0.49 | -0.56 | -0.04 | 0.21 | 0.07 | 0.36 | 0.57 | 0.36 | -0.07 |
|  | p | 0.34 | 0.26 | 0.19 | 0.94 | 0.64 | 0.88 | 0.43 | 0.18 | 0.43 | 0.88 |
| Albumin | Rho | -0.39 | -0.13 | 0.12 | -0.32 | -0.62 | -0.46 | 0.23 | 0.23 | -0.31 | -0.15 |
|  | p | 0.39 | 0.79 | 0.80 | 0.48 | 0.14 | 0.30 | 0.62 | 0.62 | 0.50 | 0.74 |
| sE-selectin | Rho | 0.14 | -0.02 | -0.38 | 0.11 | 0.29 | 0.21 | 0.14 | 0.14 | 0.64 | 0.57 |
|  | p | 0.76 | 0.97 | 0.40 | 0.81 | 0.53 | 0.64 | 0.76 | 0.76 | 0.12 | 0.18 |
| PECAM-1 | Rho | 0.07 | 0.08 | -0.02 | -0.07 | 0.18 | 0.14 | -0.25 | 0.14 | 0.50 | 0.11 |
|  | p | 0.88 | 0.87 | 0.97 | 0.87 | 0.70 | 0.76 | 0.59 | 0.76 | 0.25 | 0.82 |
| Pentraxin-3 | Rho | -0.04 | -0.20 | -0.23 | -0.26 | -0.14 | -0.18 | 0.21 | 0.43 | 0.71 | 0.71 |
|  | p | 0.94 | 0.67 | 0.61 | 0.57 | 0.76 | 0.70 | 0.64 | 0.34 | 0.07 | 0.07 |
| Tissue Factor | Rho | 0.39 | 0.18 | -0.02 | 0.56 | 0.57 | 0.46 | 0.00 | -0.50 | -0.25 | 0.14 |
|  | p | 0.38 | 0.70 | 0.97 | 0.20 | 0.18 | 0.29 | 1.00 | 0.25 | 0.59 | 0.76 |
| Thrombomodulin | Rho | 0.14 | -0.57 | -0.85 | -0.19 | 0.04 | -0.14 | 0.54 | 0.54 | 0.79 | 0.57 |
|  | p | 0.76 | 0.18 | 0.02 | 0.69 | 0.94 | 0.76 | 0.22 | 0.22 | 0.04 | 0.18 |

Abbreviations: CAC, coronary artery calcification; eGFR_cys-Cr_, estimated glomerular filtration rate estimated using serum creatinine and cystatin C; T50, time for half-maximal transformation from priary to secondary calciprotein particles (CPP); sE-selectin, soluble endothelial leukocyte adhesion molecule-1; PECAM-1, platelet endothelial cell adhesion molecule-1
