# Supplementary material for: 18Fluorine-sodium fluoride positron emission tomography to evaluate arterial calcification in patients with chronic kidney disease: a pilot study
Source: Int J Cardiovasc Imaging. Author manuscript; Available in PMC 2025 Nov 20. (PMC12628283; doi:10.1007/s10554-025-03553-0)
Supplement: Supplementary file 1 [file NIHMS2123082-supplement-supplement.pdf]

SUPPLEMENTARY MATERIALS

Title: ^{18}Fluorine-Sodium Fluoride Positron Emission Tomography to Evaluate Arterial Calcification in Patients with Chronic Kidney Disease: A Pilot Study

Authors: Aspan M. Shokrekhuda,*[1] Rylen Stratford,*[1] Wenzhu Mowrey,[2] Na Song,[1] Kexin Zhang,[3] Sara Saini,[3] Mario T. Di Dea,[1] Gabriel Duah,[1] Zahra Karimi,[1] Nicholas E. S. Sibinga,[4,5,6] Yonatan Schwartz,[7] Jeffrey M. Levsky,[4,7] Marc R. Dweck,[8] Renee Moadel,[1] Lionel S. Zuckier,[9] Mark I. Travin,[1,4] Wei Chen[3,5,6,10]

Supplementary Figures

Figure S1. Participant flow chart

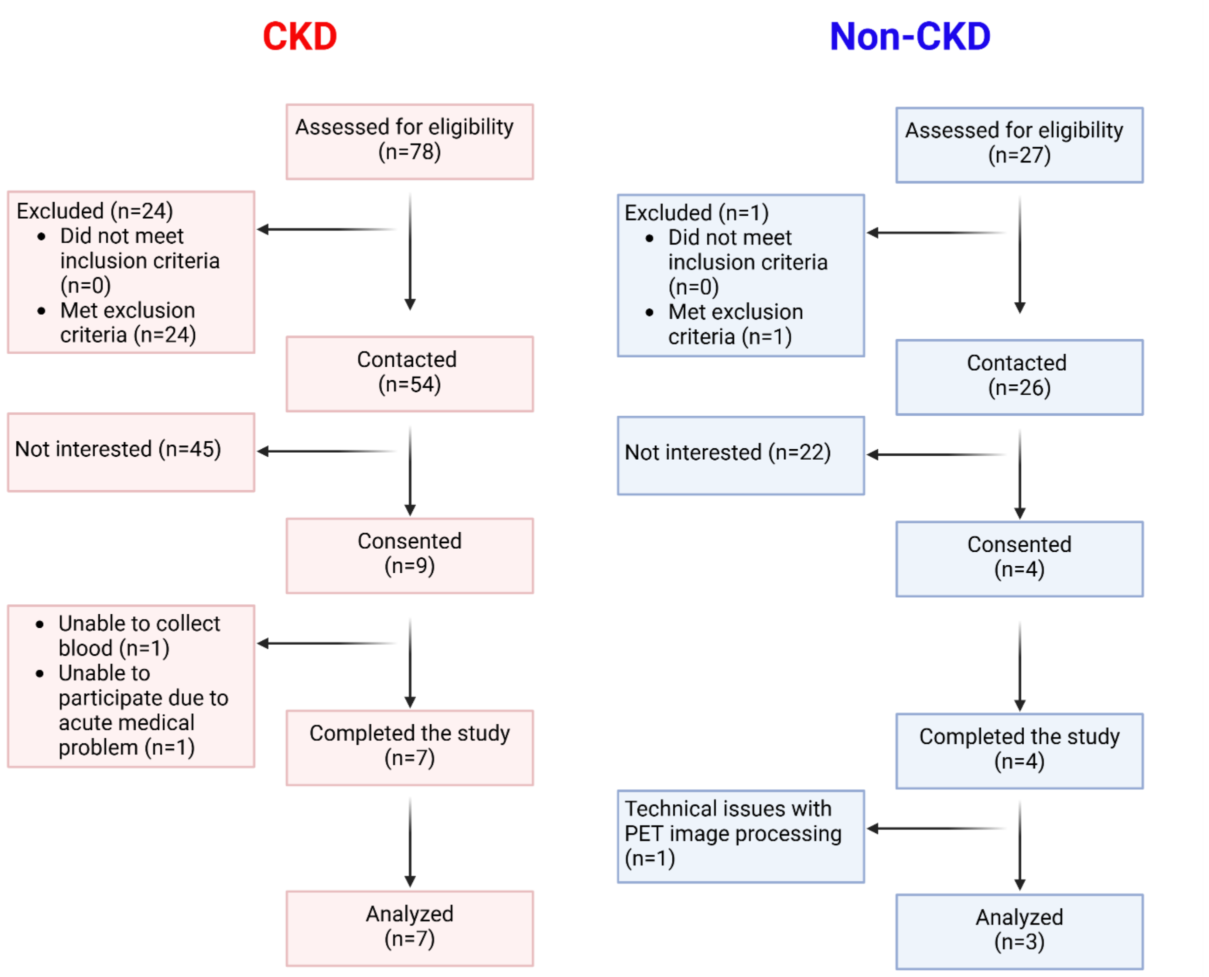

Figure S2. Volume of interest (VOIs) in aorta and reference blood pool. Aorta was divided into ascending, arch, descending thoracic, and abdominal segments. SUV_{mean} in the blood pool was obtained from right atrium and inferior vena cava (IVC).

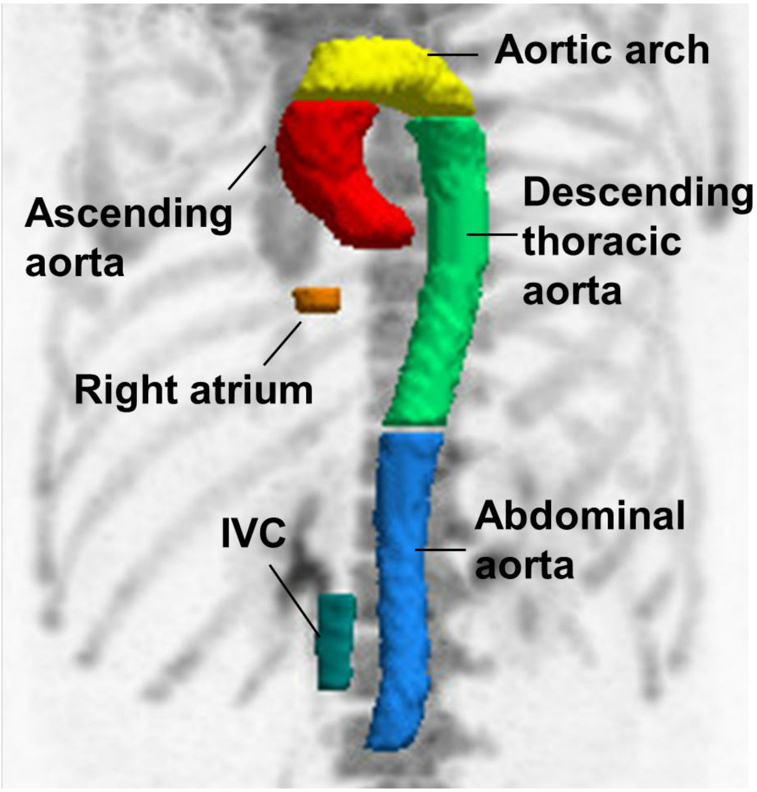

Figure S3. Macro-calcification normalized by volume of interest (VOI; mL) in different aortic segments

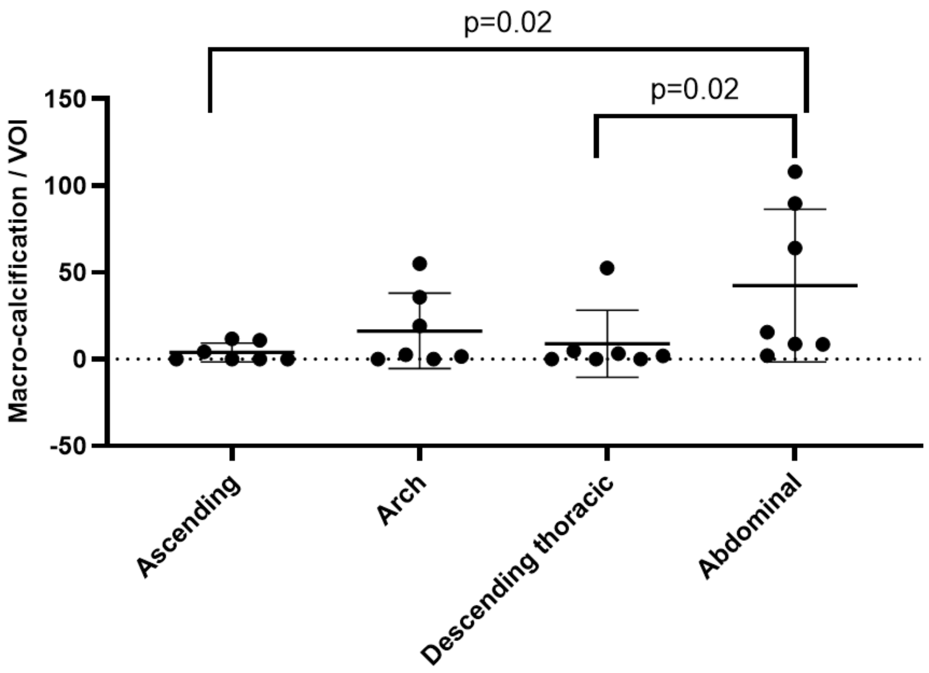

Supplementary Tables

Table S1. Correlation matrix of arterial macro-calcification and micro-calcification (in TBR$_{peak}$) among participants with CKD (n=7). Spearman correlation was used, and correlations with p<0.05 are highlighted in gray.

			Macro-calcification (CT)						Micro-calcification (PET, TBRpeak)			
			CAC	Ascending aorta	Aortic arch	Descending thoracic aorta	Abdominal aorta	Total aorta	Ascending aorta	Aortic arch	Descending thoracic aorta	Abdominal aorta
Macro-calcification (CT)	CAC	Rho	1.00									
		p	n/a									
	Ascending aorta	Rho	-0.06	1.00								
		p	0.90	n/a								
	Aortic arch	Rho	0.05	0.78	1.00							
		p	0.91	0.04	n/a							
	Descending thoracic aorta	Rho	0.70	0.59	0.39	1.00						
		p	0.08	0.16	0.38	*n/a*						
	Abdominal aorta	Rho	0.75	0.49	0.25	0.93	1.00					
		p	0.052	0.26	0.59	0.003	*n/a*					
	Total aorta	Rho	0.64	0.67	0.41	0.96	0.96	1.00				
		p	0.12	0.10	0.36	0.001	0.001	*n/a*				
Micro-calcification (PET, TBR$_{peak}$)	Ascending aorta	Rho	0.11	-0.86	-0.82	-0.35	-0.37	-0.48	1.00			
		p	0.81	0.01	0.02	0.45	0.41	0.27	*n/a*			
	Aortic arch	Rho	0.04	-0.91	-0.67	-0.59	-0.54	-0.64	0.85	1.00		
		p	0.94	0.005	0.10	0.16	0.22	0.12	0.01	*n/a*		
	Descending thoracic aorta	Rho	-0.43	-0.51	-0.68	-0.67	-0.46	-0.57	0.44	0.54	1.00	
		p	0.34	0.24	0.09	0.10	0.29	0.18	0.32	0.22	*n/a*	
	Abdominal aorta	Rho	0.00	-0.39	-0.38	-0.22	-0.25	-0.32	0.56	0.43	0.57	1.00
		p	1.00	0.38	0.40	0.63	0.59	0.48	0.20	0.34	0.18	*n/a*

Abbreviations: CAC, coronary artery calcification.

Table S2. Correlation matrix of arterial macro-calcification and micro-calcification (in TBR$_{mean}$) among participants with CKD (n=7). Spearman correlation was used, and correlations with $p<0.05$ are highlighted in gray.

			Macro-calcification (CT)						Micro-calcification (PET, TBR$_{mean}$)			
			CAC	Ascending aorta	Aortic arch	Descending thoracic aorta	Abdominal aorta	Total aorta	Ascending aorta	Aortic arch	Descending thoracic aorta	Abdominal aorta
Micro-calcification (PET, TBR$_{mean}$)	Ascending aorta	Rho	0.13	-0.37	-0.13	0.00	-0.27	-0.27	1.00			
		p	0.78	0.42	0.77	1.00	0.56	0.56	n/a			
	Aortic arch	Rho	0.15	-0.44	-0.25	-0.17	-0.24	-0.32	0.56	1.00		
		p	0.75	0.32	0.58	0.71	0.60	0.49	0.19	n/a		
	Descending thoracic aorta	Rho	0.37	-0.58	-0.42	0.00	-0.07	-0.21	0.77	0.86	1.00	
		p	0.41	0.17	0.35	1.00	0.87	0.66	0.04	0.01	n/a	
	Abdominal aorta	Rho	0.72	-0.32	-0.17	0.32	0.34	0.22	0.34	0.77	0.77	1.00
		p	0.07	0.49	0.71	0.49	0.45	0.64	0.46	0.04	0.04	n/a

Abbreviations: CAC, coronary artery calcification.

Table S3. Correlations of clinical characteristics and laboratory values with arterial macro- and micro-calcification (in TBR_{peak}) among participants with CKD (n=7). Spearman correlation was used, and correlations with p<0.05 are highlighted in gray.

		Macro-calcification (CT)						Micro-calcification (PET, TBR_{peak})			
		**CAC**	**Ascending aorta**	**Aortic arch**	**Descending thoracic aorta**	**Abdominal aorta**	**Total aorta**	**Ascending aorta**	**Aortic arch**	**Descending thoracic aorta**	**Abdominal aorta**
Age	Rho	0.63	0.59	0.59	0.80	0.85	0.85	-0.49	-0.67	-0.74	-0.58
	p	0.13	0.17	0.16	0.03	0.02	0.02	0.27	0.10	0.06	0.18
Women (Y/N)	Rho	0.47	0.09	0.64	0.16	0.16	0.16	-0.16	0.00	-0.47	-0.16
	p	0.28	0.85	0.12	0.73	0.73	0.73	0.73	1.00	0.28	0.73
Black (Y/N)	Rho	-0.61	-0.45	-0.62	-0.64	-0.61	-0.61	0.20	0.41	0.41	-0.20
	p	0.14	0.31	0.14	0.13	0.14	0.14	0.66	0.36	0.36	0.66
Diabetes mellitus (Y/N)	Rho	0.63	-0.70	-0.32	0.00	0.00	-0.16	0.79	0.63	0.00	0.47
	p	0.13	0.08	0.49	1.00	1.00	0.73	0.03	0.13	1.00	0.28
Coronary artery disease (Y/N)	Rho	0.79	0.09	0.08	0.66	0.79	0.63	0.00	-0.32	-0.32	0.00
	p	0.03	0.85	0.87	0.11	0.03	0.13	1.00	0.49	0.49	1.00
Stroke (Y/N)	Rho	0.16	-0.52	-0.24	-0.33	-0.32	-0.32	0.47	0.79	0.16	0.00
	p	0.73	0.23	0.61	0.47	0.49	0.49	0.28	0.03	0.73	1.00
Aspirin use (Y/N)	Rho	0.43	-0.72	-0.58	-0.15	0.00	-0.14	0.58	0.72	0.14	-0.14
	p	0.33	0.07	0.17	0.75	1.00	0.76	0.17	0.07	0.76	0.76
$eGFR_{cys\text{-}Cr}$	Rho	0.36	-0.49	-0.31	-0.19	0.07	-0.07	0.14	0.50	0.14	-0.36
	p	0.43	0.26	0.50	0.69	0.88	0.88	0.76	0.25	0.76	0.43
Total cholesterol	Rho	-0.86	0.02	0.11	-0.70	-0.68	-0.64	-0.46	-0.14	0.29	-0.11
	p	0.01	0.97	0.82	0.08	0.09	0.12	0.29	0.76	0.53	0.82
High-density lipoprotein	Rho	-0.90	0.38	0.29	-0.49	-0.56	-0.38	-0.58	-0.23	0.18	-0.23
	p	0.01	0.40	0.53	0.27	0.19	0.40	0.18	0.61	0.70	0.61
Low-density lipoprotein	Rho	-0.79	-0.32	-0.09	-0.82	-0.89	-0.86	-0.04	0.21	0.21	-0.04
	p	0.04	0.49	0.85	0.03	0.01	0.01	0.94	0.64	0.64	0.94
Serum calcium	Rho	0.36	0.58	0.65	0.69	0.52	0.58	-0.31	-0.74	-0.81	-0.22
	p	0.43	0.18	0.12	0.09	0.23	0.18	0.50	0.06	0.03	0.64
Serum phosphorous	Rho	0.00	0.06	-0.13	0.24	0.14	0.09	0.13	-0.38	-0.13	0.29
	p	1.00	0.90	0.79	0.60	0.76	0.85	0.79	0.40	0.79	0.53

Table S3. [continued]

		Macro-calcification (CT)						Micro-calcification (PET, TBR_{peak})			
		CAC	Ascending aorta	Aortic arch	Descending thoracic aorta	Abdominal aorta	Total aorta	Ascending aorta	Aortic arch	Descending thoracic aorta	Abdominal aorta
Serum magnesium	Rho	-0.09	-0.85	-0.92	-0.51	-0.45	-0.58	0.84	0.84	0.71	0.62
	p	0.85	0.01	0.004	0.24	0.31	0.17	0.02	0.02	0.07	0.14
Parathyroid hormone	Rho	-0.43	-0.47	-0.77	-0.59	-0.36	-0.46	0.18	0.46	0.93	0.29
	p	0.34	0.28	0.04	0.16	0.43	0.29	0.70	0.29	0.003	0.53
25-vitamin D	Rho	0.11	-0.06	-0.16	0.21	0.11	0.14	0.14	-0.02	-0.49	-0.67
	p	0.82	0.90	0.73	0.66	0.82	0.76	0.76	0.97	0.27	0.10
T_{50}	Rho	-0.36	-0.08	0.02	-0.22	-0.46	-0.32	0.14	0.14	-0.36	-0.43
	p	0.43	0.87	0.97	0.63	0.29	0.48	0.76	0.76	0.43	0.34
Secondary CPP size	Rho	0.43	-0.49	-0.56	-0.04	0.21	0.07	0.36	0.57	0.36	-0.07
	p	0.34	0.26	0.19	0.94	0.64	0.88	0.43	0.18	0.43	0.88
Albumin	Rho	-0.39	-0.13	0.12	-0.32	-0.62	-0.46	0.23	0.23	-0.31	-0.15
	p	0.39	0.79	0.80	0.48	0.14	0.30	0.62	0.62	0.50	0.74
sE-selectin	Rho	0.14	-0.02	-0.38	0.11	0.29	0.21	0.14	0.14	0.64	0.57
	p	0.76	0.97	0.40	0.81	0.53	0.64	0.76	0.76	0.12	0.18
PECAM-1	Rho	0.07	0.08	-0.02	-0.07	0.18	0.14	-0.25	0.14	0.50	0.11
	p	0.88	0.87	0.97	0.87	0.70	0.76	0.59	0.76	0.25	0.82
Pentraxin-3	Rho	-0.04	-0.20	-0.23	-0.26	-0.14	-0.18	0.21	0.43	0.71	0.71
	p	0.94	0.67	0.61	0.57	0.76	0.70	0.64	0.34	0.07	0.07
Tissue Factor	Rho	0.39	0.18	-0.02	0.56	0.57	0.46	0.00	-0.50	-0.25	0.14
	p	0.38	0.70	0.97	0.20	0.18	0.29	1.00	0.25	0.59	0.76
Thrombomodulin	Rho	0.14	-0.57	-0.85	-0.19	0.04	-0.14	0.54	0.54	0.79	0.57
	p	0.76	0.18	0.02	0.69	0.94	0.76	0.22	0.22	0.04	0.18

Abbreviations: CAC, coronary artery calcification; $eGFR_{cys\text{-}Cr}$, estimated glomerular filtration rate estimated using serum creatinine and cystatin C; T50, time for half-maximal transformation from priary to secondary calciprotein particles (CPP); sE-selectin, soluble endothelial leukocyte adhesion molecule-1; PECAM-1, platelet endothelial cell adhesion molecule-1
